# Functions and Mechanisms of SAC Phosphoinositide Phosphatases in Plants

**DOI:** 10.3389/fpls.2021.803635

**Published:** 2021-12-16

**Authors:** Yanbo Mao, Shutang Tan

**Affiliations:** MOE Key Laboratory for Cellular Dynamics, School of Life Sciences, Division of Molecular and Cell Biophysics, Division of Life Sciences and Medicine, Hefei National Science Center for Physical Sciences at the Microscale, University of Science and Technology of China, Hefei, China

**Keywords:** phosphoinositides, SAC phosphatases, subcellular localization, trafficking, *Arabidopsis*

## Abstract

Phosphatidylinositol (PtdIns) is one type of phospholipid comprising an inositol head group and two fatty acid chains covalently linked to the diacylglycerol group. In addition to their roles as compositions of cell membranes, phosphorylated PtdIns derivatives, termed phosphoinositides, execute a wide range of regulatory functions. PtdIns can be phosphorylated by various lipid kinases at 3-, 4- and/or 5- hydroxyls of the inositol ring, and the phosphorylated forms, including PtdIns3P, PtdIns4P, PtdIns5P, PtdIns(3,5)P_2_, PtdIns(4,5)P_2_, can be reversibly dephosphorylated by distinct lipid phosphatases. Amongst many other types, the SUPPRESSOR OF ACTIN (SAC) family of phosphoinositide phosphatases recently emerged as important regulators in multiple growth and developmental processes in plants. Here, we review recent advances on the biological functions, cellular activities, and molecular mechanisms of SAC domain-containing phosphoinositide phosphatases in plants. With a focus on those studies in the model plant *Arabidopsis thaliana* together with progresses in other plants, we highlight the important roles of subcellular localizations and substrate preferences of various SAC isoforms in their functions.

## Introduction

The biological membrane doesn’t only separate the cell from the outer environment, but also defines specific territory for subcellular compartments. It is mainly composed of sterols, sphingolipids and structural glycerophospholipids together with regulatory phospholipids including phosphatidylinositol and its phosphorylated derivatives phosphatidylinositol phosphates (PtdInsPs, also referred to as phosphoinositides) (Ejsing et al., 2009; Andreyev et al., 2010; Colin and Jaillais, 2020). Despite representing a small fraction of total phospholipids, these negatively charged phosphoinositides play a vital role in various cellular activities, including membrane trafficking and cellular dynamics (Gerth et al., 2017; Noack and Jaillais, 2020). Different phosphoinositides are enriched in different membranes and execute specific cellular functions (Noack and Jaillais, 2020). By application of fluorescent probes, PtdIns3P was found to distribute at multivesicular bodies/late endosomes/prevacuolar compartment (MVB/LE/PVC) and the vacuolar membrane (Vermeer et al., 2006; Simon et al., 2014); PtdIns(3,5)P_2_ localizes predominantly in MVB/LE (Hirano et al., 2017) and PtdIns(4,5)P_2_ mainly at the plasma membrane (PM; Van Leeuwen et al., 2007). These subcellular localizations may vary according to distinct cell types. In *Arabidopsis* root hairs, PtdIns(3,5)P_2_ localizes to the PM along the shank region and is involved in the hardening of the shank, whereas PtdIns(4,5)P_2_ was observed to accumulate at the apex and it is required for tip growth (Hirano et al., 2018). In root epidermal cells, PtdIns4P exhibited a gradient distribution from the highest concentration at the PM, intermediate concentration in post-Golgi/endosomal compartments to the lowest level in Golgi (Simon et al., 2014). Although both PtdIns(4,5)P_2_ and PtdIns4P accumulate at the PM, they contribute differently to PM surface charges, which give rise to different electrostatic filed along the membrane (Simon et al., 2016; Dubois and Jaillais, 2021). It is exactly these special biophysiochemical properties that determine membrane specificity and thus contribute to the organellar identity (Noack and Jaillais, 2017, 2020; Platre et al., 2018). In addition, these PtdInsPs can also recruit different lipid-binding proteins, ensuring their subcelluar localizations and functions at local membranes.

In eukaryotes, phosphoinositides can be interconverted through corresponding modifying enzymes, phosphoinositide kinases and phosphatases respectively. In plants, phosphoinositide phosphatases can be categorized into three major families, the PHOSPHATASE AND TENSIN homologs (PTEN) family, 5-Phosphatases (5-PTases) and Suppressor of Actin (SAC) domain containing phosphatases (hereafter SAC phosphatases) (Gerth et al., 2017). Here, we summarize and discuss the recent advances on this SAC family, especially the subcellular localization, substrate specificity, as well as their interacting proteins and regulators.

## Overview of Plant SAC Family

**FIGURE 1:**
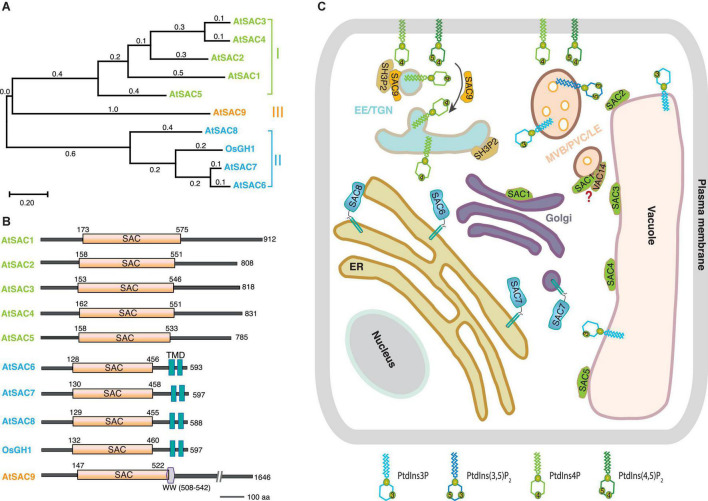
The current overview of plant SACs.**(A)** Phylogenetic analysis and **(B)** schematic domain structures of the experimentally characterized plant SACs. The phylogenetic tree was conducted by MEGA X10.2.4 with a maximum likelihood method and a Poisson correction model. The aligned protein sequences were based on 10 plant SAC members, AtSAC1 (At1g22620.1), AtSAC2 (At3g14205.1), AtSAC3 (At3g43220.1), AtSAC4 (At5g20840.1), AtSAC5 (At1g17340.1), AtSAC6 (At5g66020.1), AtSAC7 (At3g51460.1), AtSAC8 (At3G51830.1), AtSAC9 (At3g59770.3), and OsGH1 (Os02g0554300). Domain structures were determined by using the PROSITE program (https://prosite.expasy.org/). Abbreviations, SAC, suppressor of actin domain; TMD, transmembrane domain; WW, a domain with two conserved tryptophan residues. The numbers denote the amino acid residue positions. **(C)** Schematic representation of sub-cellular distribution of SACs and their putative substrates in the plant cell. AtSAC1 (green) localizes at the Golgi, whereas all the other members (AtSAC2∼AtSAC5) from subgroup I locate in the tonoplast. AtSAC6∼AtSAC8 (blue) from subgroup II are all integrated on ER through two transmembrane motifs. Additionally, AtSAC7 also localizes in post-Golgi secretory compartments. The subclade III member AtSAC9 (orange) localizes in a subpopulation of TGN/EEs close to cell cortex and thus also regulates the distribution pattern of PtdIns(4,5)P_2_. Meanwhile, AtSAC9 interacts and colocalizes with the endocytic component SH3P2. AtSAC1 colocalizes with VAC14 in MVB/LE/PVC in pollen, but it is still unclear if they can form a complex or not in plants. Abbreviations: TGN/EE, trans-Golgi network/early endosome; ER, endoplasmic reticulum; MVB/LE/PVC, multivesicular body/late endosome/pre-vacuolar compartments. The black arrow indicates the conversion of PtdIns(4,5)P_2_ to PtdIns4P during endocytosis regulated by AtSAC9.

The SAC phosphatase Sac1p was first identified in a genetic screen for the suppressor of actin defective mutant in yeast. The loss of Sac1p led to increased sensitivity to cold treatment, disorganized intracellular actin as well as decreased secretion rate in yeast (Novick et al., 1989), whereas absence of *SAC1* resulted in embryonic and preimplantation lethality in *Drosophila* and mice (Wei et al., 2003; Liu et al., 2008). In yeast and mammals, SAC domain proteins can be further divided into two subfamilies. Members of the first subfamily contain the SAC domain and the type II phosphatidylinositol phosphatase 5-phosphatase domain, including yeast Inp51p, Inp52p, and Inp53p, and mammalian synaptojanin1 and synaptojanin2. The other subfamily includes yeast Sac1p and Fig4p, and mammalian counterparts Sac1, Sac2/INPP5f, and Sac3 (Hughes et al., 2000a; Manford et al., 2010). Based on sequence homology, nine SAC phosphatases (SAC1∼SAC9) have been found in both *Arabidopsis thaliana* and rice (*Oryza sativa* L.) genome, respectively, forming three subgroups (Table 1 and Figure 1A; Zhong and Ye, 2003; Novakova et al., 2014): (I) AtSAC1 ∼ AtSAC5 form a clade with medium protein size, exhibiting high similarity to yeast Fig4p (Zhong et al., 2005; Novakova et al., 2014). (II) The Sac1p closest homologs AtSAC6/AtSAC1b, AtSAC7/ROOT HAIR DEFFECTIVE4 (RHD4)/NON-CYTOLEDON PHENOTYPE2 (NCP2)/AtSAC1c, and AtSAC8/AtSAC1a all contain the two transmembrane domains (TMD) at their C-termini (Despres et al., 2003; Zhong and Ye, 2003; Thole et al., 2008). Notably, ectopic expression of *AtSAC6* to *AtSAC8* rescued the cold-sensitive phenotype associated with ablation of yeast Sac1p (Despres et al., 2003). The recently characterized rice GRAIN NUMBER AND PLANT HEIGHT (OsGH1) was also classified into this subclade according to its protein size and domain organization (Guo et al., 2020; Figure 1B). (III) The third clade only contains one member, AtSAC9, which possesses a larger protein size and a unique protein sequence, a WW protein-protein interaction domain following the N-terminal SAC catalytic domain plus around 1,100 amino acid residues at the C-terminus with unknown function (Zhong and Ye, 2003; Williams et al., 2005; Figure 1B).

**TABLE 1:** Subcellular localization and substrate specificity of currently characterized plant SAC family members.

Subgroup	Name	Localization	Putative substrate	References
Clade I	AtSAC1/FRA7	Golgi apparatus	PtdIns(3,5)P_2_	Zhong et al., 2005
	AtSAC2/3/4/5	Tonoplast	PtdIns(3,5)P_2_	Novakova et al., 2014
Clade II	AtSAC6/AtSAC1b	Endoplasmic reticulum	PtdIns4P, PtdIns(4,5)P_2_	Despres et al., 2003; Song et al., 2021
	AtSAC7/RHD4/NCP2/AtSAC1c	Endoplasmic reticulum	PtdIns4P, PtdIns(4,5)P_2_	Despres et al., 2003; Thole et al., 2008; Song et al., 2021
	AtSAC8/AtSAC1a	Endoplasmic reticulum	PtdIns4P, PtdIns(4,5)P_2_	Despres et al., 2003; Song et al., 2021
	OsGH1	Endoplasmic reticulum	PtdIns4P, PtdIns(4,5)P_2_	Guo et al., 2020
Clade III	AtSAC9	Subpopulation of trans-Golgi network/early endosomes	Ins(1,4,5)P_3_, PtdIns(4,5)P_2_	Williams et al., 2005; Doumane et al., 2021

## Substrate Specificity

The SAC domain is highly conserved and profiled with PtdInsP phosphatase activity among eukaryotes (Del Bel and Brill, 2018). *In vitro* biochemical studies revealed that mouse Sac1 and yeast Sac1p are capable of dephosphorylating PtdIns3P, PtdIns4P and PtdIns(3,5)P_2_. Whereas the levels of PtdIns3P only slightly increased (around 1.5-fold) in yeast *sac1* mutant, 8- to 10-fold elevated levels of PtdIns4P were detected in this mutant (Guo et al., 1999; Hughes et al., 2000b; Nemoto et al., 2000). These data indicated that PtdIns4P, rather than PtdIns3P, might be a preferential substrate for Sac1p, and that enzyme specificities do not always correlate between *in vitro* and *in vivo* assays. This could be due to the differences in the subcellular distributions of phospholipids and SAC1 enzymes, or potential additional components involved in the catalysis *in vivo*.

In *Arabidopsis*, AtSAC1 exhibited phosphatase activity toward PtdIns(3,5)P_2_ as observed by an *in vitro* activity assay (Zhong et al., 2005). Overexpression of *AtSAC2*∼*AtSAC5* resulted in the reduction of PtdIns(3,5)P_2_, whereas the level of PtdIns(3,5)P_2_ was unchanged in the *sac3 sac4 sac5* triple mutant, perhaps due to a compensatory response. Nevertheless, together with the abundance of PtdIns3P in the tonoplast, it is implied that AtSAC2∼AtSAC5 are responsible for the conversion of PtdIns(3,5)P_2_ to PtdIns3P (Zhong et al., 2005; Novakova et al., 2014). Overall, AtSAC1∼AtSAC5 share high similarity to the homologous yeast Fig4p on the phosphatase activity toward PtdIns(3,5)P_2_ (Rudge et al., 2004; Duex et al., 2006).

In line with the *in vitro* data that AtSAC7 displayed a preference for PtdIns4P, around a 50% increase of PtdIns4P was detected in the root of the *sac7* mutant compared to wild type (Thole et al., 2008). By use of an ion chromatography system, both PtdIns4P and PtdIns(4,5)P_2_ contents were found to dramatically increase in *sac6/7*, *sac6/8* and *sac7/8* double mutants, while a slight increase was detected in single mutants (Song et al., 2021), suggesting that PtdIns4P and PtdIns(4,5)P_2_ might be the substrates for AtSAC6/7/8. In rice, the close related homolog OsGH1 specifically dephosphorylated PtdIns4P and PtdIns(4,5)P_2_ from both *in vitro* phosphatase activity assay and binding studies. Moreover, the *OsGH1* knockout plant showed a significant increase in endogenous PtdIns4P and PtdIns(4,5)P_2_ levels, whereas *OsGH1-*overexpressing plants exhibited reduced PtdIns4P and PtdIns(4,5)P_2_ levels (Guo et al., 2020), differently from the yeast homolog Sac1p and mammalian SAC1, indicating the functional diversity among the eukaryotes. These findings suggested that the plant SAC family clade II displayed phosphatase activity toward PtdIns4P and PtdIns(4,5)P_2_. In addition, AtSAC9 was also reported to act as a PtdIns(4,5)P_2_ phosphatase based on the observation of approximately 4-fold higher PtdIns(4,5)P_2_ levels in root extracts of loss-of-function *sac9* mutant (Williams et al., 2005). Nevertheless, the elevated level of Ins(1,4,5)P_3_ was also detected in *sac9* (Williams et al., 2005), indicating the catalytic diversity of AtSAC9. The overall substrate preference is summarized here based on the previous research (Table 1), but more direct biochemical evidence is still required to validate the substrate specificity for each SAC enzyme.

## The Expression Pattern and Subcellular Localization of SACs

In *Arabidopsis*, transcripts of all the nine *AtSACs* were detected in most tissues (Despres et al., 2003; Zhong and Ye, 2003; Song et al., 2021), implying pleotropic functions in various life activities. Among the members of subgroup I, AtSAC1 was reported to colocalize with the Golgi marker AVP2-ECFP when co-expressed in carrot protoplasts. The truncation of its C terminus by the *fra7* mutation resulted in its mislocalization at the cytoplasm, but the phosphatase catalytic activity was unaffected. These results suggest that the sub-cellular localization of AtSAC1 might be essential for its biological functions (Zhong et al., 2005). Interestingly, in pollen and pollen tubes, over-expression of AtSAC1-GFP under the *LAT52* promoter was found to reside at the PVC (Zhang et al., 2018), implying the subcellular distribution pattern may vary in distinct cell types. Furthermore, AtSAC2∼AtSAC5 were all observed to localize in the tonoplast, and this thus ensures the abundance of PtdIns3P in the tonoplast for appropriate maintenance of vacuolar morphology (Novakova et al., 2014). Similarly, the yeast homolog Fig4p was also identified to localize in the limiting membrane of yeast vacuoles, controlling the level of PtdIns(3,5)P_2_ upon hyperosmotic shock (Rudge et al., 2004), suggesting a conserved role of SACs from this subgroup in vacuole development among eukaryotes.

In yeast, Sac1p is an integral protein residing at endoplasmic reticulum (ER) and Golgi apparatus membrane, and regulates the PtdIns4P pool that is important for vacuolar morphology and Golgi trafficking (Whitters et al., 1993; Foti et al., 2001). The endoplasmic reticulum localization was also identified for mammalian and *Drosophila* Sac1 (Nemoto et al., 2000; Liu et al., 2008; Forrest et al., 2013). Consistently, the rice homolog OsGH1 was reported to co-localize with the ER marker in rice protoplasts. Disruption of OsGH1 destroyed homeostasis of membrane PtdIns4P and PtdIns(4,5)P_2_, thus affecting organelle morphology and cell development (Guo et al., 2020). In *Arabidopsis*, AtSAC6/7/8 targeted to the ER compartment when expressed in tobacco BY2 cells together with a C-terminally fused GFP (Despres et al., 2003), and they are required for both the maintenance of PtdIns(4,5)P_2_ polarity and the restriction of PtdIns4P at PM in root hairs (Thole et al., 2008; Song et al., 2021). Intriguingly, the N-terminal fusion version EYFP-AtSAC7 which is functional, as demonstrated by rescuing the phenotype of the *sac7* loss-of-function mutant, localized at the post-Golgi secretory compartments and regulated the accumulation of PtdIns4P on membrane compartments at the tips of growing root hairs (Thole et al., 2008). Future experimental validations are thus needed to address whether all these three SAC isoforms share the same subcellular localization. A recent study has shown that AtSAC9 localizes to a subpopulation of trans-Golgi network/early endosomes in close vicinity to the PM in meristematic epidermal cells of *Arabidopsis* roots, restricting the distribution of its substrate PtdIns(4,5)P_2_ at the PM for proper regulation of endocytosis (Doumane et al., 2021; Table 1 and Figure 1C).

## Functions and Mechanisms of SACs and Phosphoinositides

The anionic lipids, PtdIns4P, PtdIns(3,5)P_2_, and PtdIns(4,5)P_2_, regulate many cellular processes, including endocytosis, vacuolar trafficking and actin dynamics, though in low abundance (Gerth et al., 2017; Hirano and Sato, 2019; Noack and Jaillais, 2020). Deficiency in these phosphoinositides or dysfunction of their related metabolic enzymes SAC phosphatases usually causes defects in endocytic trafficking and results in abnormal vacuole, Golgi and endosome morphology (Rudge et al., 2004; Liu et al., 2008; Whitley et al., 2009; He et al., 2017). In *Arabidopsis*, the *AtSAC1-*truncated mutant *fra7* (*fragile fiber7*) displayed a wide range of defects in plant growth and architecture, including a decrease of cell wall thickness and cell length in fiber cells and vessel elements, and aberrant actin organization (Zhong et al., 2005). However, how loss of *AtSAC1* and accumulation of PtdIns(3,5)P_2_ are related with these various phenotypes is still elusive. Interestingly, seedlings overexpressing *AtSAC2* ∼*AtSAC5* or the *sac2 sac3 sac4 sac5* quadruple mutant and *sac3 sac4 sac5* triple mutant all exhibited arrested growth on medium without sucrose. It is proposed that fragmented vacuoles and abnormal protein trafficking toward the vacuole in the *sac* mutants might explain for the decreased viability in seedlings (Novakova et al., 2014). In yeast, the vacuole-localized Fig4p physically associates with an adaptor-like protein Vac14p in FAB1/PIKfyve protein complex and thus regulates the biosynthesis and turnover of PtdIns(3,5)P_2_, controlling the vacuolar size (Rudge et al., 2004; Duex et al., 2006; Jin et al., 2008). Moreover, Vac14 can also control Fig4p localization in yeast (Rudge et al., 2004), indicating that the subcellular distribution of the phosphatase is directed by its interacting partner. AtSAC1 was also found to colocalize with the yeast Vac14p homolog VAC14 at PVC in wortmannin (an inhibitor of PI3K)-treated pollen tubes in *Arabidopsis* (Zhang et al., 2018).

Consistent with the functional redundancy among the SAC family, the single mutant of *sac6*, *sac7*, and *sac8* didn’t show any obvious defects in plant development except the short-bulged root hairs in *sac7*/*rhd4-1* (Schiefelbein and Somerville, 1990; Thole et al., 2008). However, in the double and triple mutants, significantly delayed embryonic development or even lethal embryos were observed, correlated with a decreased auxin distribution (Song et al., 2021). Furthermore, the retrograde trafficking of auxin efflux carrier PIN-FORMED1 (PIN1) and PIN2 proteins was also affected in the loss of *AtSAC7/8*, suggesting a role of AtSAC6-AtSAC8 in auxin-mediated development (Song et al., 2021). In rice, over-accumulation of PtdIns4P and PtdIns(4,5)P_2_ caused by dysfunction of *OsGH1* disrupted the activity of actin-related protein 2/3 (Arp2/3) complex for actin nucleation, thus resulting in the reduced plant height, panicle and grain size. In addition, the morphology of Golgi apparatus and chloroplast was also perturbed by elevated PtdInsP4 and PtdIns(4,5)P_2_ levels (Guo et al., 2020), highlighting the conserved role of PtdInsP homeostasis in organellar organization in rice. In *Drosophila*, Vesicle-associated membrane protein (VAMP)-Associated Protein (DVAP) physically interacts with SAC1 in controlling phosphoinositide metabolism (Forrest et al., 2013), providing the evidence for the potential working mechanism of SACs in vesicle trafficking.

In *Arabidopsis*, the endocytic component Src Homology 3 Domain Protein 2 (SH3P2) was identified as a AtSAC9-interacting partner through yeast two-hybrid screening. Absence of AtSAC9 resulted in mis-localization of PtdIns(4,5)P_2_, and also triggered the altered SH3P2 localization and reduced clathrin-mediated endocytosis rate (Doumane et al., 2021). Moreover, *sac9* knock-out mutant exhibited cell wall defects and overall stressed phenotype, including dwarfism, closed stomata, anthocyanin accumulation, and increased transcription of stress response genes (Williams et al., 2005; Vollmer et al., 2011). These results suggest that the AtSAC9 phosphatase is required for modulating phosphoinositide distribution during stress response and cell wall deposition.

## Conclusion

In plants, three types of phosphoinositides, PtdIns4P, PtdIns(3,5)P_2_, and PtdIns(4,5)P_2_, have been identified so far as substrates of SAC phosphatases within distinct endomembrane compartments. Malfunction of SACs severely disrupts PtdInsP homeostasis reflected on both contents and distribution patterns, thus affecting endomembrane trafficking and organellar organization, and results in a wide range of developmental defects. However, it is still less understood how SACs execute their catalytic functions and associate with membrane in the case of those isoforms lacking TMDs. Future studies might be required to determine the regulatory molecules and interacting partners to better understand cellular pathways in which SACs are involved.

## Author Contributions

YM and ST conceptualized and wrote the manuscript. YM made the figures. Both authors contributed to the article and approved the submitted version.

## Conflict of Interest

The authors declare that the research was conducted in the absence of any commercial or financial relationships that could be construed as a potential conflict of interest.

## Publisher’s Note

All claims expressed in this article are solely those of the authors and do not necessarily represent those of their affiliated organizations, or those of the publisher, the editors and the reviewers. Any product that may be evaluated in this article, or claim that may be made by its manufacturer, is not guaranteed or endorsed by the publisher.
